# Impact of Body Composition and Physical Function on Quality of Life After Gastrectomy for Gastric Cancer

**DOI:** 10.3389/fsurg.2021.832351

**Published:** 2022-01-20

**Authors:** Wen-Bin Wang, Hao-Nan Song, Dong-Dong Huang, Xin Luo, Hui-Yang Cai, Jing-Yi Yan, Wei-Zhe Chen, Chun-Gen Xing, Qian-Tong Dong, Xiao-Lei Chen

**Affiliations:** ^1^Department of Gastrointestinal Surgery, The First Affiliated Hospital of Wenzhou Medical University, Wenzhou, China; ^2^Department of General Surgery, The Second Affiliated Hospital of Soochow University, Suzhou, China

**Keywords:** handgrip strength (HGS), gait speed, subcutaneous fat area (SFA), visceral fat area (VFA), skeletal muscle density (SMD)

## Abstract

**Purpose:**

Patients with gastric cancer after gastrectomy often suffer from a decline in their quality of life (QoL), but the relationship between body composition (BC) and physical function on QoL has rarely been studied. This study aims to evaluate and determine the changes in QoL after gastrectomy and the impact of BC and physical function on QoL.

**Methods:**

A total of 311 gastric cancer patients completed EORTC QLQ-C30 and EORTC QLQ-STO22 questionnaires before and 1, 3, 6 months post-surgery. Data including BC, handgrip strength (HGS) and 6-m gait speed (GS) were collected prospectively. Multiple linear regression analysis was used to determine the correlation between QoL and BC, HGS and GS.

**Results:**

Patients had significantly worse scores after surgery on most function and symptom scales (*p* < 0.001), but most of these scales recovered within 6 months after surgery. A higher subcutaneous fat area (SFA)was associated with increased symptom scores 1 month after surgery. A higher GS is associated with a better global health status symptom.

**Conclusion:**

Patients suffer from a decline in their QoL after gastrectomy for gastric cancer. Intervention strategies aiming at reducing SFA and improving GS may improve the QoL in patients underwent gastrectomy for gastric cancer.

## Introduction

Gastric cancer is the fifth most common malignant tumor and the fourth leading cause of cancer death in the world (1). The incidence of gastric cancer is highest in East Asia (2). Although the treatment of tumor has made great progress, the main treatment for resectable gastric tumor is surgical resection with or without adjuvant chemotherapy (3). However, gastrectomy has a negative impact on the quality of life (QoL) (4–6). Dissatisfaction with life of patients after gastrectomy is mostly diet-related symptoms, including reflux, early satiety, nausea and pain (7).Therefore, medical workers and researchers are paying more and more attention to improving the QoL of survivors (8). So far, many studies on QoL after gastrectomy have been reported, most of which focused on the relationship between gender, age, type of reconstruction, the extent of gastric resection, surgical approach, and complications and QoL (9–16). Weight loss and changes in body composition (BC) are very common after gastrectomy, and these changes will affect the QoL (17). Nutritional assessment based on BC measurement can reflect BC, metabolic characteristics and physiological reserves, and is a factor that determines prognosis (18). The patient's 6-meter gait speed (GS) has always been used as an indicator of poor prognosis (19–21). However, few studies have reported the correlation between BC, physical function and QoL after gastrectomy.

The purpose of this study is to describe the changes in the patient's QoL within 6 months after gastrectomy, and to clarify the relationship between preoperative handgrip strength (HGS), GS, BC and QoL at 1, 3, and 6 months after surgery.

## Materials and Methods

### Patients

This study included patients who underwent gastric cancer surgery in the First Affiliated Hospital of Wenzhou Medical University from 2014 to 2020. The research protocol has been approved by the Ethical Review Committee of the First Affiliated Hospital of Wenzhou Medical University. This project has been registered in the China Clinical Trial Registration Center (NO. ChiCTR1800019717). Inclusion criteria: (1) Age ≥18 years; (2) American Society of Anesthesiologists (ASA) score ≤3; (3) Preoperative gastroscopy pathology suggests gastric adenocarcinoma; (4) CT examination of the abdomen in our hospital within 1 month before the operation; (5) have complete questionnaire on the QoL before and within 6 months after surgery; (6) Agreed to participate in this study and signed an informed consent.

Exclusion criteria: (1) Incurable tumor; (2) The patient who cannot cooperate with the measurement of HGS or GS; (3) The patient has received radiotherapy or chemotherapy before surgery.

### Data Collection

The following data were prospectively collected (1) Preoperative patient demographic and disease characteristics, including gender, age, body mass index (BMI), ASA score grade, previous history of abdominal surgery. (2) Surgical details: including laparoscopic assisted surgery, type of gastrectomy, type of reconstruction. (3) Pathological data: the surgical specimens are tested by the pathology department to obtain the pathological type, tumor location, degree of differentiation, and tumor-node-metastasis (TNM) staging (according to the TNM staging of the 8th edition of American Joint Committee on Cancer (AJCC) (22).

### Hand Grip Strength and Physical Function

When the patient was admitted to the hospital, the patients were taught to use the maximum strength of the dominant hand to grasp the electronic grip (model: EH101; CAMRY, Guangdong, China) and take the average of two measurements (23–26).

The GS of 6 meters test is often used to evaluate as a physical function. The patients were taught to walk 6 meters at a normal speed without assistance, we record the required time and take the average of 2 measurements.

### Quality of Life Assessment

Patients complete the QoL questionnaire after admission of hospital and during the routine follow-up after surgery. Postoperative follow-up was completed in three time periods: 1, 3, and 6 months after surgery. The main purpose of this follow-up is to assess the recovery of the QoL after gastrectomy over time, so only those patients who completed all preoperative and postoperative follow-ups were included in the analysis.

QLQ-C30 and QLQ-STO22 are adopted in QoL assessment. The EORTC QLQ-C30 questionnaire is used to assess the QoL of patients with cancer in general (27). EORTC QLQ-STO22 is specially developed to evaluate the HRQL of gastric cancer patients (28). According to the conversion formula provided by EORTC, the questionnaire score can be linearly converted into 0 to 100 points.

The EORTC QLQ-C30 scale can be divided into 1 global QoL scale, 5 functional scales (physical function, role function, emotional function, cognitive function, social function) and 9 symptom subscales (fatigue, nausea and vomiting, pain, dyspnea, insomnia, appetite loss, constipation, diarrhea, financial difficulties). QLQ-STO22 can be divided into 9 symptom subscales (dysphagia, pain, reflux, eating restriction, anxiety, dry mouth, body image, hair loss, taste loss). The functional scale and the general health status scale are positive score scales, the higher the score, the better the QoL; the symptom scale are negative score scales, the higher the score, the worse the QoL.

### Measurement Body Composition Parameters

By analyzing CT images at the level of the third lumbar vertebra (L3), using image processing system (GE ADW 4.5), BC parameters were measured to determine skeletal muscle and abdominal adipose tissue area [subcutaneous fat area (SFA) and visceral fat area (VFA)]. Tissue Hounsfield Unit (HU)thresholds were as follows: skeletal muscle −29 to +150 HU, SFA −190 to −0 HU, VFA −150 to −50 HU (29). Skeletal muscle was normalized for height in meters squared to calculate the skeletal muscle index (SMI). Skeletal muscle density (SMD) was identified by the average value of HU in the muscle area. In order to explore the distribution of abdominal fat tissue, we also calculated the visceral fat area to subcutaneous fat area ratio (VSR).

### Statistical Analysis

K-S test is used for normality test, The continuity data of the normal distribution is expressed as: mean and standard deviation (SD), and the non-normal distribution continuity data was expressed as: the median and the interquartile range (IQR). QoL 1, 3, 6 months after surgery were compared with preoperative QoL by using the paired Wilcoxon test. Finally, multivariable linear regression analysis with stepwise backward elimination was performed to evaluate the association between QoL scales and HGS, GS, BC paramenters, demographic characteristics variables. A *P*-value <0.05 is considered statistically significant. All statistical analyses were performed using IBM SPSS version 22.

## Results

### Patient Characteristics

A total of 311 patients were included in the final analysis in this study. Human body baselines are shown in Table 1. Of the 311 individuals included, 227(73.0%) were men. The average HGS and GS of all patients were 27.6 ± 9.1 kg and 1.0 ± 0.2 m/s. The average body mass index (BMI) was 22.6 ±3.0 kg/m^2^. The median of SMD and SMI are 37.2 HU and 43.7 cm^2^/m^2^, respectively. The median of SFA and VFA are 0.9 and 0.9 dm^2^, respectively. The median age at diagnosis was 64 years. One hundred and fifteen (37.0%) patients underwent total gastrectomy, 101 (32.5%) patients underwent laparoscopy-assisted operation. Among all patients, 119 (38.3%) patients used Billroth I for gastrointestinal reconstruction, 40 (12.9%) patients used Billroth II method, and the remaining 152 (48.9%) used Roux-en-Y method. The majority of patients (*n* = 204, 65.6%) had an advanced tumor stage (≥II).

**Table 1:** Baseline characteristics of the patients.

**Factor**	***n =* 311 (%)**
Age, median (IQR), years	64.0(15.0)
BMI, mean (SD),kg/m^2^	22.6(3.0)
HGS, mean (SD), kg	27.6(9.1)
GS, mean (SD), m/s	1.0(0.2)
L3 SMD, median (IQR), HU	37.2(10.6)
L3 SMI, median (IQR), cm^2^/m^2^	43.7(10.3)
SFA, median (IQR), dm^2^	0.9(0.7)
VFA, median (IQR), dm^2^	0.9(0.9)
VSR, median (IQR)	0.98(0.81)
**Gender**	
Male	227(73.0)
Female	84(27.0)
**ASA**	
I	102(32.8)
II	156(50.2)
III	53(17.0)
**Type of gastrectomy**	
Distal gastrectomy	196(63.0)
Total gastrectomy	115(37.0)
**Laparoscopy-assisted operation**	
Yes	101(32.5)
No	210(67.5)
**TNM stage**	
0	0(0)
I	107(34.4)
II	80(25.7)
III	124(39.9)
**Type of reconstruction**	
Billroth I	119(38.3)
Billroth II	40(12.9)
Roux-en-Y	152(48.9)
**Pathological type**	
Differentiated	92(29.6)
Undifferentiated	219(70.4)
**Tumor location**	
Proximal	43(13.8)
Middle	65(20.9)
Distal	200(64.3)
Linitis plastica	3(1)
**Previous abdominal surgery**	
Yes	36(11.6)
No	275(88.4)

*SD, standard deviation; IQR, interquartile range; BMI, body mass index; HGS, handgrip strength; GS: gait speed; ASA, American Society of Anesthesiologists; SMD, skeletal muscle density; SMI, skeletal muscle index; SFA, subcutaneous fat area; VFA, visceral fat area; VSR, VFA to SFA ratio; TNM, tumor-node-metastasis*.

*The values in the table were number of patients unless indicated otherwise*.

### Postoperative QoL vs. Preoperative QoL

The average QoL score and standard deviation of the study population were shown in Table 2. In the first month after the operation, all functional scales except for the emotional function decreased compared to before surgery, and then gradually increased within 6 months after surgery. At 6 months after the operation, the scores of role function, emotional function and cognitive function returned to the preoperative level, while the scores of physical function and social function have not yet recovered to the preoperative level. The score of global health status continued to improve after surgery, and was significantly higher than before surgery at 6 months after surgery.

**Table 2:** Mean scores of EORTC QLQ-C30 and QLQ-STO22 scales.

	**Preoperative** **QoL**	***P*-value^**c**^**	**1 month after surgery QoL**	***P*-value^**d**^**	**3 months after surgery QoL**	***P*-value^**e**^**	**6 months after** **surgery QoL**
**Functional scales** ^**a**^							
Physical	92.6(6.0)	**<0.001**	77.4(13.7)	**<0.001**	82.4(12.6)	**<0.001**	87.5(11.8)
Role	88.8(9.0)	**<0.001**	76.1(19.4)	**0.013**	86.4(18.4)	0.116	91.0(14.8)
Emotional	92.1(5.1)	**<0.001**	93.8(11.6)	**<0.001**	96.4(9.3)	**<0.001**	97.8(6.6)
Cognitive	98.2(3.5)	**0.004**	96.0(8.7)	**<0.001**	97.7(7.6)	**<0.001**	99.2(3.5)
Social	92.6(8.8)	**<0.001**	78.7(18.8)	**<0.001**	79.0(18.7)	**<0.001**	84.7(16.9)
**General symptom scales** ^**b**^							
Fatigue	13.0(8.4)	**<0.001**	26.1(18.1)	**<0.001**	21.5(18.9)	0.241	15.8(16.9)
Nausea and vomiting	3.5(6.6)	**<0.001**	5.0(14.2)	0.080	10.8(18.2)	**<0.001**	6.1(14.2)
Pain	8.3(6.3)	0.369	8.9(12.3)	**<0.001**	5.5(10.2)	**<0.001**	3.5(8.5)
Dyspnoea	3.9(6.2)	**<0.001**	4.1(12.7)	**<0.001**	1.4(6.7)	**<0.001**	1.0(5.6)
Insomnia	10.9(10.9)	**0.008**	9.5(17.7)	**<0.001**	6.8(15.6)	**0.010**	4.3(11.8)
Appetite loss	11.5(12.2)	0.266	15.2(22.8)	**<0.001**	16.4(21.0)	0.799	12.4(19.4)
Constipation	3.2(6.8)	**<0.001**	2.2(11.6)	**<0.001**	2.7(10.9)	**<0.001**	1.0(6.8)
Diarrhea	1.6(4.1)	**<0.001**	5.2(13.0)	**<0.001**	3.9(10.7)	**<0.001**	3.7(10.9)
Financial difficulties	2.6(5.1)	**<0.001**	3.6(11.0)	**<0.001**	1.6(7.6)	**<0.001**	1.1(5.9)
Global health status^a^	62.5(7.6)	**0.041**	63.7(16.3)	**<0.001**	71.5(17.1)	**<0.001**	78.7(17.3)
**QLQ-STO22**							
**General symptom scales** ^**b**^							
Dysphagia	10.2(6.1)	0.922	13.8(14.5)	**<0.001**	9.6(7.1)	**<0.001**	6.6(6.2)
Pain	13.2(7.3)	**<0.001**	8.5(10.9)	**<0.001**	8.0(11.8)	**<0.001**	3.4(8.3)
Reflux	7.2(8.1)	**0.001**	6.2(10.7)	**<0.001**	6.2(11.7)	**<0.001**	4.2(9.4)
Eating restrictions	1.6(2.7)	**0.001**	4.3(10.7)	**<0.001**	2.9(7.5)	**<0.001**	2.0(5.7)
Anxiety	12.4(8.5)	**<0.001**	10.3(14.6)	**<0.001**	5.8(13.0)	**<0.001**	2.5(6.6)
Dry mouth	22.8(9.8)	**<0.001**	12.6(18.7)	**<0.001**	8.2(15.1)	**<0.001**	4.7(11.9)
Taste	4.8(7.3)	**<0.001**	8.7(20.9)	**<0.001**	7.0(17.7)	**<0.001**	4.8(14.0)
Body image	1.3(3.7)	**<0.001**	3.0(9.9)	**<0.001**	1.2(8.2)	**<0.001**	0.4(3.8)
Hair loss	1.1(3.1)	**<0.001**	0.6(3.6)	**<0.001**	2.4(8.2)	**<0.001**	1.7(7.1)

*Scores are presented as mean [± SD]*.

a *Score range 0–100: higher scores represent a better quality of life or level of functioning*.

b *Score range 0–100: higher scores represent more severe symptoms*.

c *Preoperative QoL compared with 1 month after surgery QoL*.

d *Preoperative QoL compared with 3 months after surgery QoL*.

e *Preoperative QoL compared with 6 months after surgery QoL*.

*Bold values indicate significant variables (p < 0.05)*.

On General symptom scales, most symptoms become worse after surgery. In addition, pain, appetite loss, and dysphagia symptom scale scores were not statistically different before and 1 month after surgery, indicating that these symptoms did not change after surgery at this point in time. At 6 months after surgery, nausea and vomiting, diarrhea, eating restrictions, hair loss symptom scales scores were still higher than those before surgery. The scores of the rest of the symptom scales all showed a gradual decline and improved significantly.

### Predictive Factors for QoL 1, 3, 6 Months After Surgery

Table 3 shows that SFA is associated with poor QoL at 1 month after surgery, such as nausea and vomiting [4.2 (2.7; 5.6), *p* < 0.001], appetite loss [4.8 (2.4; 7.3), *p* < 0.001], constipation [4.1 (2.9; 5.3), *p* < 0.001], dysphagia [2.3 (0.7; 3.8), *p* = 0.004], global health status [−2.5 (−4.4; −0.5), *p* = 0.014]. VFA is negatively correlated with role function [−5.1 (−9.6; −0.6), *p* = 0.028] and cognitive function [−2.7 (−4.9; −0.6), *p* = 0.013], but it is positively correlated with anxiety [−4.5 (−8.0; −1.0), *p* = 0.012]. GS is positively correlated with insomnia [−12.1 (−22.2; −2.0), *p* = 0.02], global health status [12.7 (2.6; 22.9), *p* = 0.014] and hair loss [−2.4 (−4.6; −0.2), *p* = 0.032], but it is also a poor social function [−18.3 (−28.9; −7.8), *p* = 0.001] significant predictor.

**Table 3:** Multivariable linear regression model on quality of life, symptom scales, and functional scales from EORTC QLQ-C30 and QLQ-STO22 for 1 month after surgery.

	**Physical functioning^**a**^**	**Role functioning^**a**^**	**Emotional functioning^**a**^**	**Cognitive functioning^**a**^**	**Social functioning^**a**^**	**Fatigue^**b**^**	**Nausea and vomiting^**b**^**	**Pain^**b**^**	**Dyspnea^**b**^**	**Insomnia^**b**^**	**Appetite loss^**b**^**	**Constipation^**b**^**	**Diarrhea^**b**^**	**Financial problems^**b**^**	**Global health status^**a**^**
**Quality of life from the EORTC QLQ-C30 questionnaire**															
Gender	–	–	–	–	–	–	–	–	–	–	–	–	5.0 (0.4;9.6)	–	–
Age	–	–	–	–	–	–	−0.2 (−0.3;0.0)	–	–	–	–	–	–	–	–
BMI	–	1.3(0.2;2.4)	–	0.6(0.0;1.1)	–	–	–	–	−0.9 (−1.5;−0.3)	–	–	−0.9 (−1.4;−0.5)	–	–	–
ASA	−3.8 (−6.2;−1.3)	−5.0 (−8.4;−1.6)	–	–	–	–	–	2.7 (0.6;4.9)	3.6 (1.2;5.9)	3.3 (0.2;6.4)	–	–	3.4 (1.1;5.8)	–	–
SFA	–	–	–	–	–	–	4.2 (2.7;5.6)	–	–	–	4.8 (2.4;7.3)	4.1(2.9;5.3)	–	–	−2.5 (−4.4;−0.5)
VFA	–	−5.1 (−9.6;−0.6)	–	−2.7 (−4.9;−0.6)	–	–	–	–	–	–	–	–	–	–	–
VSR	–	–	–	–	–	–	–	–	–	–	–	–	–	–	–
SMD	–	–	–	–	–	–	–	–	–	–	–	–	–	–	–
SMI	–	–	–	–	–	–	–	–	0.3 (0.0;0.5)	–	–	–	–	–	–
HGS	–	–	–	–	–	−0.3 (−0.6;0.0)	–	–	–	–	–	–	−0.3 (−0.5;0.0)	–	−0.3 (−0.5;−0.1)
GS	–	–	–	–	−18.3 (−28.9;−7.8)	–	–	–	–	−12.1 (−22.2;−2.0)	–	–	–	–	12.7 (2.6;22.9)
Tumor location	–	–	–	–	4.0 (1.0;7.0)	–	–	−2.8 (−5.4;−0.3)	−2.9 (−5.6;−0.1)	–	–	–	–	–	–
Pathological type	–	–	–	–	–	–	–	–	–	–	–	–	3.8(0.4;7.3)	–	–
TNM stage	–	–	–	–	–	–	–	–	–	–	–	–	–	–	–
Type of reconstruction	–	–	−1.9 (−3.6;−0.1)	–	–	–	–	–	–	–	–	−1.6 (−3.1;−0.2)	–	–	−2.3 (−4.3;−0.3)
Laparoscopy–assisted operation	–	–	–	−2.4 (−4.7;−0.1)	–	–	–	–	–	–	–	–	–	–	–
Type of gastrectomy	–	–	–	–	–	–	–	−4.0 (−7.9;−0.1)	−6.1 (−10.2;−1.9)	–	–	–	–	−3.3 (−6.1;−0.5)	–
Previous abdominal surgery	–	–	–	–	–	–	–	–	–	–	–	–	–	–	6.5 (0.5;12.5)
		**Dysphagia** ^**b**^		**Pain** ^**b**^	**Reflux** ^**b**^		**Eating restrictions** ^**b**^		**Anxiety** ^**b**^	**Dry mouth** ^**b**^		**Taste** ^**b**^	**Body image** ^**b**^		**Hair loss** ^**b**^
**Quality of life from the EORTC QLQ-STO22 questionnaire**															
Gender		–		–	–		–		–	–		–	–		–
Age		–		–	–		–		–	–		–	0.1(0.0;0.2)		–
BMI		–		–	–		–		–	−1.2(-2.1;-0.2)		–	–		–
ASA		–		–	–		–		3.0(0.5;5.6)	4.5(1.1;7.9)		–	–		–
SFA		2.3(0.7;3.8)		–	–		–		–	–		–	–		–
VFA		–		–	–		–		−4.5(-8.0;-1.0)	–		–	–		–
VSR		–		–	–		–		–	–		–	–		–
SMD		–		–	–		–		−0.4(-0.7;-0.2)	−0.5(-0.8;-0.1)		–	–		–
SMI		0.3(0.0;0.5)		–	−0.2(-0.4;0.0)		–		–	–		–	–		–
HGS		–		−0.2(-0.3;0.0)	–		–		–	–		–	–		–
GS		–		–	–		–		–	–		–	–		−2.4(-4.6;-0.2)
Tumor location		−2.3(-4.6;0.0)		–	–		–		–	–		–	−1.7(-3.4;0.0)		−0.8(-1.5;-0.1)
Pathological type		–		–	–		–		–	5.0(0.1;9.8)		–	–		–
TNM stage		–		–	–		–		–	–		–	−1.5(-2.9;-0.1)		–
Type of reconstruction		–		–	–		–		–	–		–	–		−0.8(-1.4;-0.2)
Laparoscopy-assisted operation		–		3.9(1.2;6.7)	–		–		–	–		–	–		–
Type of gastrectomy		–		–	–		–		–	–		–	–		–
Previous abdominal surgery		7.3(1.9;12.6)		–	–		–		–	–		–	–		–

*Scores are presented as linear regression coefficients, with 95% confidence intervals between brackets. During stepwise backward linear regression, the weakest associated variables and Insignificant variables (p > 0.05) are excluded from the model (–)*;

a *Higher scores represent better quality of life or functioning*;

b *Higher scores represent more symptoms*.

It can be seen from Table 4 that VFA is a predictor of good QoL at 3 months after surgery. It is positively correlated with cognitive functioning [1.4 (0.0; 2.7), *p* = 0.045], dyspnea [−2.3 (−3.9; −0.7), *p* = 0.005], global health status [3.1 (0.4; 5.8), *p* = 0.027] and eating restrictions [−1.8 (−3.5; −0.1), *p* = 0.042]. VSR is positively correlated with social functioning [3.3 (0.2; 6.5), *p* = 0.039], but patients with high VSR have symptoms of dyspnea [1.9 (0.2; 3.6), *p* = 0.028], eating restrictions [2.2 (0.3; 4.1), *p* = 0.021]. High GS is an independent predictor of better QoL scores for cognitive functioning [6.3 (1.2; 11.3), *p* = 0.016], financial problems [−5.6 (−10.6; −0.5), *p* = 0.032], reflux [−7.6 (−14.2; −1.0), *p* = 0.023]. At the same time, it is also a predictor of hair loss [7.1 (1.9; 12.3), *p* = 0.007]. SMI is associated with higher nausea and vomiting [0.4 (0.1; 0.8), *p* = 0.014], pain [0.3 (0.1; 0.6), *p* = 0.004] and anxiety [0.4 (0.1; 0.7), *p* = 0.009] scores.

**Table 4:** Multivariable linear regression model on quality of life, symptom scales, and functional scales from EORTC QLQ-C30 and QLQ-STO22 for 3 months after surgery.

	**Physical functioning^**a**^**	**Role functioning^**a**^**	**Emotional functioning^**a**^**	**Cognitive functioning^**a**^**	**Social functioning^**a**^**	**Fatigue^**b**^**	**Nausea and vomiting^**b**^**	**Pain^**b**^**	**Dyspnea^**b**^**	**Insomnia^**b**^**	**Appetite loss^**b**^**	**Constipation^**b**^**	**Diarrhea^**b**^**	**Financial problems^**b**^**	**Global health status^**a**^**
**Quality of life from the EORTC QLQ-C30 questionnaire**															
Gender	4.9 (1.6;8.2)	–	–	3.1 (0.4;5.8)	–	–	−9.5 (−15.5;−3.5)	–	−4.1 (−6.6;−1.7)	–	−6.8 (−12.4;−1.2)	−4.0 (−7.6;−0.4)	–	–	–
Age	–	–	–	–	–	–	–	–	–	–	–	−0.1 (−0.3;0.0)	−0.2 (−0.3;0.0)	−0.1 (−0.2;0.0)	−0.3 (−0.5;−0.1)
BMI	–	–	–	–	–	–	–	−0.5 (−1.0;0.0)	–	−0.7 (−1.3;0.0)	–	−1.0 (1.7;−0.2)–	–	−0.4 (−0.7;−0.1)	–
ASA	–	–	–	–	–	–	–	3.9 (2.0;5.7)	1.6 (0.3;2.9)	–	–	–	2.2 (0.2;4.1)	3.0 (1.5;4.4)	–
SFA	–	–	–	–	–	–	–	–	–	–	–	–	–	–	–
VFA	–	–	–	1.4 (0.0;2.7)	–	–	–	–	−2.3 (−3.9;−0.7)	–	–	–	–	–	3.1 (0.4;5.8)
VSR	–	–	–	–	3.3 (0.2;6.5)	–	–	–	1.9 (0.2;3.6)	–	–	–	–	–	–
SMD	–	–	–	–	–	–	–	–	–	–	–	–	–	–	–
SMI	–	–	–	–	–	–	0.4 (0.1;0.8)	0.3 (0.1;0.6)	–	–	–	–	–	–	–
HGS	–	–	–	–	–	–	–	–	–	–	–	–	–	–	–
GS	–	–	–	6.3 (1.2;11.3)	–	–	–	–	–	–	–	–	–	−5.6 (−10.6;−0.5)	–
Tumor location	–	–	–	–	–	–	–	–	–	–	–	–	–	–	–
Pathological type	–	–	–	–	–	–	–	–	–	4.3 (0.1;8.5)	–	–	–	–	–
TNM stage	–	−3.2 (−5.7;−0.7)	–	–	–	–	3.3 (0.8;5.9)	–	–	–	–	–	–	–	–
Type of reconstruction	–	–	–	–	–	–	–	–	–	–	2.7 (0.0;5.4)	–	–	–	–
Laparoscopy–assisted operation	–	–	–	–	–	–	–	–	–	–	–	–	–	–	–
Type of gastrectomy	–	–	–	–	−6.9 (−12.8;−1.0)	4.8 (0.3;9.4)	–	−5.1 (−8.8;−1.3)	–	–	–	–	–	–	–
Previous abdominal surgery	–	–	–	–	–	–	7.2 (0.1;14.3)	–	–	–	–	–	–	–	–
		**Dysphagia** ^**b**^		**Pain** ^**b**^	**Reflux** ^**b**^		**Eating restrictions** ^**b**^		**Anxiety** ^**b**^	**Dry mouth** ^**b**^		**Taste** ^**b**^	**Body image** ^**b**^	**Hair loss** ^**b**^	
**Quality of life from the EORTC QLQ-STO22 questionnaire**															
Gender		–		–	–		−3.2 (−5.5;−0.8)		−7.3 (−11.5;−3.1)	−5.5 (−10.5;−0.5)		–	–	–	
Age		–		0.2 (0.0;0.3)	–		–		–	–		–	–	–	
BMI		–		–	–		–		−1.0 (−1.7;−0.4)	−0.9 (−1.7;−0.1)		–	–	–	
ASA		–		–	–		–		–	4.1 (1.3;6.9)		–	1.6 (0.1;3.1)	–	
SFA		–		–	–		–		–	–		–	–	–	
VFA		–		–	–		−1.8 (−3.5;−0.1)		–	–		–	–	–	
VSR		–		–	–		2.2 (0.3;4.1)		–	–		–	–	–	
SMD		–		–	–		–		–	–		0.3 (0.0;0.6)	–	–	
SMI		–		–	–		–		0.4 (0.1;0.7)	–		–	–	–	
HGS		–		–	–		–		–	–		–	–	−0.2 (−0.3;0.0)	
GS		–		–	−7.6 (−14.2;−1.0)		–		–	–		–	–	7.1 (1.9;12.3)	
Tumor location		–		–	–		–		–	–		–	–	–	
Pathological type		–		−3.3(−6.2;−0.4)	−4.2(−7.2;−1.2)		–		–	–		–	–	–	
TNM stage		–		–	–		–		–	–		2.9(0.4;5.4)	–	–	
Type of reconstruction		–		–	–		–		–	–		–	–	–	
Laparoscopy–assisted operation		–		–	–		–		–	–		–	–	–	
Type of gastrectomy		–		–	–		2.0(0.1;3.9)		–	–		–	–	–	
Previous abdominal surgery		–		–	–		–		–	–		–	–	–	

*Scores are presented as linear regression coefficients, with 95% confidence intervals between brackets. During stepwise backward linear regression, the weakest associated variables and Insignificant variables (p>0.05) are excluded from the model (–)*;

a *Higher scores represent better quality of life or functioning*;

b *Higher scores represent more symptoms*.

The multiple linear regression model at 6 months after surgery was shown in Table 5. VSR is an independent predictor of better global health status [3.1 (0.1; 6.1), *p* = 0.04]. SFA and insomnia [2.2 (0.9;3.4), *p* = 0.001] are negatively correlated. A higher VFA is negatively correlated with nausea and vomiting [3.0 (0.7; 5.3), *p* = 0.01]. GS is positively correlated with emotional functioning [5.6 (0.9; 10.4), *p* = 0.02], cognitive functioning [2.8 (0.6; 5.0), *p* = 0.014], reflux [−5.5 (−11.0; −0.1), *p* = 0.046]. However it is negatively correlated with social functioning [– 14.3 (−24.8; −3.7), *p* = 0.008]. SMD is a good predictor for fatigue [−0.4 (−0.7; −0.2), *p* = 0.002], appetite loss [−0.4 (−0.7; −0.1), *p* = 0.011], anxiety [−0.2 (−0.3; −0.1), *p* = 0.001] and dry mouth [−0.2 (−0.4;0.0), *p* = 0.037]. At the same time, it is also a predictor of higher dyspnea [0.1 (0.0;0.2), *p* = 0.017] QoL score.

**Table 5:** Multivariable linear regression model on quality of life, symptom scales, and functional scales from EORTC QLQ-C30 and QLQ-STO22 for 6 months after surgery.

	**Physical functioning^**a**^**	**Role functioning^**a**^**	**Emotional functioning^**a**^**	**Cognitive functioning^**a**^**	**Social functioning^**a**^**	**Fatigue^**b**^**	**Nausea and vomiting^**b**^**	**Pain^**b**^**	**Dyspnea^**b**^**	**Insomnia^**b**^**	**Appetite loss^**b**^**	**Constipation^**b**^**	**Diarrhea^**b**^**	**Financial problems^**b**^**	**Global** **health** **status^**a**^**
**Quality of life from the EORTC QLQ-C30 questionnaire**															
Gender	3.7 (0.5;6.8)	–	−2.8 (−5.3;−0.4)	–	–	–	–	–	−2.1 (−3.6;−0.5)	–	−5.4 (−10.7;−0.1)	–	–	–	–
Age	−0.1 (−0.3;0.0)	−0.2 (−0.3;0.0)	0.1 (0.0;0.2)	0.1 (0.0;0.1)	–	–	–	–	–	–	–	–	–	–	–
BMI	–	–	−0.3 (−0.6;0.0)	–	–	–	–	–	–	–	–	–	–	−0.3 (−0.5;0.0)	–
ASA	–	–	–	–	–	–	–	2.1 (0.6;3.6)	–	–	–	–	–	–	–
SFA	–	–	–	–	–	–	–	–	–	2.2 (0.9;3.4)	–	–	–	–	–
VFA	–	–	–	–	–	–	3.0 (0.7;5.3)	–	–	–	–	–	–	–	–
VSR	–	–	–	–	–	–	–	–	–	–	–	–	–	–	3.1 (0.1;6.1)
SMD	–	–	–	–	–	−0.4 (−0.7;−0.2)	–	–	0.1 (0.0;0.2)	–	−0.4 (−0.7;−0.1)	–	–	–	–
SMI	–	0.3 (0.0;0.5)	–	–	–	–	–	–	–	–	–	–	–	–	–
HGS	–	–	–	–	–	–	−0.2 (−0.4;−0.1)	–	–	–	–	–	–	–	–
GS	–	–	5.6 (0.9;10.4)	2.8 (0.6;5.0)	−14.3 (−24.8;−3.7)	–	–	–	–	–	–	–	–	–	–
Tumor location	–	–	−1.3 (−2.4;−0.2)	–	–	–	3.2 (0.3;6.1)	–	–	–	–	–	4.0 (1.7;6.3)	–	–
Pathological type	–	–	–	–	–	–	–	–	–	–	–	–	–	1.7 (0.1;3.3)	–
TNM stage	–	–	–	–	–	–	–	1.4 (0.1;2.7)	–	–	–	–	–	–	–
Type of reconstruction	–	–	–	0.7 (0.2;1.1)	–	–	–	–	–	–	–	–	–	–	–
Laparoscopy–assisted operation	–	–	–	–	–	4.6 (0.4;8.9)	–	–	–	–	–	–	–	–	–
Type of gastrectomy	–	–	–	–	–	–	–	−4.0 (−6.8;−1.1)	–	–	–	–	–	–	–
Previous abdominal surgery	–	–	–	–	–	–	–	–	–	–	–	–	–	–	–
		**Dysphagia** ^**b**^		**Pain** ^**b**^	**Reflux** ^**b**^		**Eating restrictions** ^**b**^		**Anxiety** ^**b**^	**Dry mouth** ^**b**^		**Taste** ^**b**^	**Body image** ^**b**^		**Hair loss** ^**b**^
**Quality of life from the EORTC QLQ-STO22 questionnaire**															
Gender		–		–	–		–		–	–		–	–		–
Age		0.1 (0.0;0.1)		–	–		–		–	–		0.2 (0.0;0.3)	–		–
BMI		0.3 (0.1;0.6)		–	–		–		–	–		–	–		–
ASA		–		–	–		1.1 (0.1;2.1)		–	–		–	–		–
SFA		–		–	–		–		–	–		–	–		–
VFA		–		–	–		–		–	–		–	–		–
VSR		–		–	–		–		–	–		–	–		–
SMD		–		–	–		–		−0.2 (−0.3;−0.1)	−0.2 (−0.4;0.0)		–	–		–
SMI		–		–	–		–		–	–		–	–		–
HGS		–		–	–		–		–	–		–	–		–
GS		–		–	−5.5 (−11.0;−0.1)		–		–	–		–	–		–
Tumor location		–		–	−2.5 (−4.5;−0.4)		–		–	–		–	–		–
Pathological type		–		–	−2.7 (−5.2;−0.2)		–		–	–		–	–		−2.2 (−3.8;−0.6)
TNM stage		−0.9 (−1.8;0.0)		–	–		–		–	–		–	–		–
Type of reconstruction		–		–	–		–		–	–		–	–		–
Laparoscopy–assisted operation		–		–	−2.8 (−5.3;−0.4)		–		–	–		–	–		–
Type of gastrectomy		1.9 (0.3;3.5)		–	–		–		–	–		–	–		–
Previous abdominal surgery		–		–	–		–		–	–		–	–		–

*Scores are presented as linear regression coefficients, with 95% confidence intervals between brackets. During stepwise backward linear regression, the weakest associated variables and Insignificant variables (p > 0.05) are excluded from the model (–)*;

a *Higher scores represent better quality of life or functioning*;

b *Higher scores represent more symptoms*.

## Discussion

In the present study, we investigated the relationship between CT-based BC parameters, physical function and QoL 1, 3, 6 months after surgery in patients with gastric cancer. To the best of our knowledge, this is the first prospective study to investigate these correlations in gastric cancer. Our study showed that gastrectomy had a significant negative impact on QoL (5). However, most functional and symptomatic damages recovered within 6 months. These findings were similar to the results of another study by Hu et al. (5, 30). The results of multiple linear regression showed that patients with higher GS at the time of diagnosis had better global health status scores 1 month after surgery. In addition, higher pace is associated with lower symptom score. In the first month after surgery, patients with a higher SFA at diagnosis usually had a poorer QoL, mainly manifested by a higher score on the symptom scale. Three and six months after surgery, SFA has no significant relationship with QoL. At 1 month after surgery, higher VFA content is associated with deterioration of role functioning and cognitive functioning, and it is associated with the reduction of appetite loss and anxiety symptoms. At 3 months after surgery, VFA has a protective effect on some functions, symptom scales and global health status. At 6 months after surgery, VSR is positively correlated with global health status. HGS, SMI and SMD showed minimal correlation with postoperative QoL. In addition, on some scales, males and lower ASA scores are predictors of better QoL (11).

Similar findings regarding the importance of BC on the QoL after surgery have also been demonstrated in several previous studies. The study by Biljana Gigic et al. concluded that for patients after colorectal cancer surgery, VFA was negatively correlated with social function and pain scores at a 6-month follow-up. At 12 months after surgery, VFA was negatively correlated with body function (31).In addition, Sheean's research showed that compared with non-obese women, abdominal obese women with estrogen receptor-positive metastatic breast cancer had significantly higher levels of serum inflammation biomarkers, more severe symptoms, and lower QoL (32). Different from the conclusion of Biljana Gigic et al., our results show that high visceral fat is beneficial to the QoL at 3 months after surgery. The possible explanation is that visceral fat has a physiological role of providing energy during stress, which promotes the repair of the body (33). According to our team's previous research results, VFA is a double-edged sword for gastric cancer patients. In patients with normal BMI, the positive effect of VFA on nutritional status may exceed its negative effect (34). Therefore, for patients with normal BMI, the protective effect of VFA on QoL may be more obvious.

The studies by Swisher et al. and Gudmundsson et al. show that higher SFA has an adverse effect on postoperative QoL. In view of these studies, we can recommend cancer patients with higher SFA to actively exercise and control their diet, which can reduce their body fat rate and improve their QoL (35, 36).

In the present study, GS showed a strongest predictive effect on QoL compared with other confounding factors. For example, 1 month after surgery, GS is the strongest predictor of global health. GS, as one of the measurement methods of physical performance, is used to diagnose sarcopenia. According to the European Working Group on Sarcopenia in Older People (EWGSOP) diagnostic criteria for sarcopenia, a single cut-off speed ≤ 0.8 m/s is used as an indicator of severe sarcopenia (26). In recent years, Charlotte Beaudart and his colleagues had invented a QoL questionnaire for patients with sarcopenia and found that there was a decline in the QoL in patients with sarcopenia (37). The research of our colleagues also found that patients with sarcopenia have a lower QoL after surgery (38). Decreased GS should be regarded as a sign of poor QoL after surgery.

In clinical studies, GS has been used as an effective tool to assess the elderly who are at high risk of adverse outcomes (19–21). There is a lot of evidence that poor physical functioning is associated with readmission rates (39), disability (40, 41), mortality (19), falls and depression (19, 42).These findings indicate that low GS at diagnosis is a powerful predictor of poor QoL and adverse outcomes in patients after gastrectomy.

GS as a safe, fast and simple tool is not commonly used clinically, even though International Academy on Nutrition and Aging (IANA) research shows that the usual gait as a single item tool is as sensitive as comprehensive tools in predicting adverse outcomes (19). We can use the GS test as a routine preoperative examination like abdominal CT. In this way, the characteristics that determine the patient's short-term QoL can be identified as early as possible. Our findings indicate that patients with high SFA or low GS at diagnosis have a lower QoL after gastrectomy. Therefore, these patients may need a customized plan to reduce SFA and improve physical function. For example, in the process of tumor treatment, moderate- or vigorous-intensity exercise (43), reasonable diet, and strengthening of nutritional support will help to improve their QoL.

The advantage of our research is the use of CT to define the composition of BC, which allows us to accurately define the fat and muscle tissue of patients with gastric cancer. Secondly, CT is used as a routine preoperative examination for patients with gastric cancer, which means that there is no need for another exposure to radiation. Our main innovation is to comprehensively analyze the effects of various components of human BC and physical functions on the QoL, and conduct a multi-factor analysis on them.

This study has several limitations. First of all, this is a single-center study, and the results of the study may not be representative of the general population. Secondly, the postoperative follow-up time for patients is short, and because postoperative abdominal CT examinations are not frequent performed, postoperative BC data are missing. The long-term BC and QoL after surgery still need to be verified in future studies.

## Conclusions

This research found that patients with gastric cancer suffer from a decline in their QoL after gastrectomy. Besides, a lower gait speed or a higher subcutaneous fat area before surgery is associated with a worse quality of life after surgery. Therefore, based on current research, exercise and diet control may improve the quality of life in patients with gastric cancer after gastrectomy.

**Code availability:** SPSS software (version 22.0 IBM, Armonk, NY, USA).

## Data Availability Statement

The raw data supporting the conclusions of this article will be made available by the authors, without undue reservation.

## Ethics Statement

The studies involving human participants were reviewed and approved by the Ethical Review Committee of the First Affiliated Hospital of Wenzhou Medical University. The patients/participants provided their written informed consent to participate in this study.

## Author Contributions

X-LC: contributed to the study design and revised the article. Q-TD, C-GX, H-NS, J-YY, and H-YC: collected the data. W-ZC and D-DH: analyzed and interpreted the data. H-YC and XL: helped to draft the article. W-BW: wrote the article. All authors read and approved the final manuscript.

## Funding

This research was funded by the Zhejiang Provincial Health Department Medical Support Discipline-Nutrition (11-ZC24). Special Fund of Zhejiang Upper Gastrointestinal Tumor Diagnosis and Treatment Technology Research Center (jbzx-202006).

## Conflict of Interest

The authors declare that the research was conducted in the absence of any commercial or financial relationships that could be construed as a potential conflict of interest.

## Publisher's Note

All claims expressed in this article are solely those of the authors and do not necessarily represent those of their affiliated organizations, or those of the publisher, the editors and the reviewers. Any product that may be evaluated in this article, or claim that may be made by its manufacturer, is not guaranteed or endorsed by the publisher.

## Abbreviations

SD: standard deviation
IQR: interquartile range
BMI: body mass index
HGS: handgrip strength
GS: gait speed
ASA: American Society of Anesthesiologists
SMD: skeletal muscle density
SMI: skeletal muscle index
SFA: subcutaneous fat area
VFA: visceral fat area
VSR: VFA to SFA ratio
TNM: tumor-node-metastasis
QoL: quality of life
BC: body composition
BCMS: BC measurement
AJCC: American Joint Committee on Cancer
HU: Tissue Hounsfield Unit.

## References

[1] Sung H Ferlay J Siegel RL Laversanne M Soerjomataram I Jemal A. Global Cancer Statistics 2020: GLOBOCAN Estimates of Incidence and Mortality Worldwide for 36 Cancers in 185 Countries. CA Cancer J Clin. (2021) 71:209–49. 10.3322/caac.21660 33538338

[2] Torre LA Bray F Siegel RL Ferlay J Lortet-Tieulent J Jemal A. Global cancer statistics, 2012. CA Cancer J Clin. (2015) 65:87–108. 10.3322/caac.21262 25651787

[3] Japanese Gastric Cancer A. Japanese gastric cancer treatment guidelines 2018 (5th edition). Gastric Cancer. (2021) 24:1–21. 10.1007/s10120-020-01042-y 32060757 PMC7790804

[4] Yu W Park KB Chung HY Kwon OK Lee SS. Chronological changes of quality of life in long-term survivors after gastrectomy for gastric cancer. Cancer Res Treat. (2016) 48:1030–6. 10.4143/crt.2015.398 27004956 PMC4946352

[5] Hu Y Vos EL Baser RE Schattner MA Nishimura M Coit DG. Longitudinal analysis of quality-of-life recovery after gastrectomy for cancer. Ann Surg Oncol. (2021) 28:48–56. 10.1245/s10434-020-09274-z 33125569 PMC8354003

[6] Randle RW Swords DS Levine EA Fino NF Squires MH Poultsides G. Optimal extent of lymphadenectomy for gastric adenocarcinoma: A 7-institution study of the US gastric cancer collaborative. J Surg Oncol. (2016) 113:750–5. 10.1002/jso.24227 26996496 PMC4874863

[7] Nakada K Ikeda M Takahashi M Kinami S Yoshida M Uenosono Y. Characteristics and clinical relevance of postgastrectomy syndrome assessment scale (PGSAS)-45: newly developed integrated questionnaires for assessment of living status and quality of life in postgastrectomy patients. Gastric Cancer. (2015) 18:147–58. 10.1007/s10120-014-0344-4 24515247

[8] Lee SS Chung HY Kwon O Yu W. Long-term shifting patterns in quality of life after distal subtotal gastrectomy: preoperative- and healthy-based interpretations. Ann Surg. (2015) 261:1131–7. 10.1097/SLA.0000000000000832 25072431

[9] Han DS Ahn J Ahn HS. Are the elderly patient's changes in the health-related quality of life one year after gastrectomy for stomach cancer different from those in young patients? Ann Surg Treat Res. (2021) 100:8–17. 10.4174/astr.2021.100.1.8 33457392 PMC7791192

[10] Yabusaki H Kodera Y Fukushima N Hiki N Kinami S Yoshida M. Comparison of postoperative quality of life among three different reconstruction methods after proximal gastrectomy: insights from the PGSAS study. World J Surg. (2020) 44:3433–40. 10.1007/s00268-020-05629-5 32506229 PMC7458934

[11] Brenkman HJF Tegels JJW Ruurda JP Luyer MDP Kouwenhoven EA Draaisma WA. Factors influencing health-related quality of life after gastrectomy for cancer. Gastric Cancer. (2018) 21:524–32. 10.1007/s10120-017-0771-0 29067597 PMC5906484

[12] Namikawa T Oki T Kitagawa H Okabayashi T Kobayashi M Hanazaki K. Impact of jejunal pouch interposition reconstruction after proximal gastrectomy for early gastric cancer on quality of life: short- and long-term consequences. Am J Surg. (2012) 204:203–9. 10.1016/j.amjsurg.2011.09.035 22813641

[13] Kim YW Baik YH Yun YH Nam BH Kim DH Choi IJ. Improved quality of life outcomes after laparoscopy-assisted distal gastrectomy for early gastric cancer: results of a prospective randomized clinical trial. Ann Surg. (2008) 248:721–7. 10.1097/SLA.0b013e318185e62e 18948798

[14] Misawa K Fujiwara M Ando M Ito S Mochizuki Y Ito Y. Long-term quality of life after laparoscopic distal gastrectomy for early gastric cancer: results of a prospective multi-institutional comparative trial. Gastric Cancer. (2015) 18:417–25. 10.1007/s10120-014-0374-y 24801197

[15] Yasuda K Shiraishi N Etoh T Shiromizu A Inomata M Kitano S. Long-term quality of life after laparoscopy-assisted distal gastrectomy for gastric cancer. Surg Endosc. (2007) 21:2150–3. 10.1007/s00464-007-9322-9 17479329

[16] Park S Chung HY Lee SS Kwon O Yu W. Serial comparisons of quality of life after distal subtotal or total gastrectomy: what are the rational approaches for quality of life management? J Gastric Cancer. (2014) 14:32–8. 10.5230/jgc.2014.14.1.32 24765535 PMC3996247

[17] Katsube T Konnno S Murayama M Kuhara K Sagawa M Yoshimatsu K. Changes of nutritional status after distal gastrectomy in patients with gastric cancer. Hepatogastroenterology. (2008) 55:1864–7. 19102410

[18] Ahima RS Lazar MA. The health risk of obesity-better metrics imperative. Science. (2013) 341:856–8. 10.1126/science.1241244 23970691

[19] van KanGARollandYAndrieuSBauerJBeauchetOBonnefoyM. Gait speed at usual pace as a predictor of adverse outcomes in community-dwelling older people an international academy on nutrition and aging (Iana) task force. J Nutr Health Aging. (2009) 13:881–9. 10.1007/s12603-009-0246-z19924348

[20] Montero-Odasso M Schapira M Soriano ER Varela M Kaplan R Camera LA. Gait velocity as a single predictor of adverse events in healthy seniors aged 75 years and older. J Gerontol a-Biol. (2005) 60:1304–9. 10.1093/gerona/60.10.1304 16282564

[21] Abellan van KanGRollandYBergmanHMorleyJEKritchevskySBVellasB. The I.A.N.A Task Force on frailty assessment of older people in clinical practice. J Nutr Health Aging. (2008) 12:29–37. 10.1007/BF0298216118165842

[22] In H Solsky I Palis B Langdon-Embry M Ajani J Sano T. Validation of the 8th edition of the AJCC TNM staging system for gastric cancer using the national cancer database. Ann Surg Oncol. (2017) 24:3683–91. 10.1245/s10434-017-6078-x 28895113

[23] Maggio M Ceda GP Ticinesi A De Vita F Gelmini G Costantino C. Instrumental and non-instrumental evaluation of 4-meter walking speed in older individuals. PLoS ONE. (2016) 11:e0153583. 10.1371/journal.pone.0153583 27077744 PMC4831727

[24] Rydwik E Bergland A Forsen L Frandin K. Investigation into the reliability and validity of the measurement of elderly people's clinical walking speed: a systematic review. Physiother Theory Pract. (2012) 28:238–56. 10.3109/09593985.2011.601804 21929322

[25] Chen LK Liu LK Woo J Assantachai P Auyeung TW Bahyah KS. Sarcopenia in Asia: consensus report of the Asian Working Group for Sarcopenia. J Am Med Dir Assoc. (2014) 15:95–101. 10.1016/j.jamda.2013.11.025 24461239

[26] Cruz-Jentoft AJ Bahat G Bauer J Boirie Y Bruyere O Cederholm T. Sarcopenia: revised European consensus on definition and diagnosis. Age Ageing. (2019) 48:16–31. 10.1093/ageing/afy169 30312372 PMC6322506

[27] Aaronson N Ahmedzai S Bergman B Bullinger M Cull A Duez N. The European Organization for Research and Treatment of Cancer QLQ-C30: a quality-of-life instrument for use in international clinical trials in oncology. J Natl Cancer Inst. (1993) 85:365–76. 10.1093/jnci/85.5.365 8433390

[28] Blazeby JM Conroy T Bottomley A Vickery C Arraras J Sezer O. Clinical and psychometric validation of a questionnaire module, the EORTC QLQ-STO 22, to assess quality of life in patients with gastric cancer. Eur J Cancer. (2004) 40:2260–8. 10.1016/j.ejca.2004.05.023 15454251

[29] Fujiwara N Nakagawa H Kudo Y Tateishi R Taguri M Watadani T. Sarcopenia, intramuscular fat deposition, and visceral adiposity independently predict the outcomes of hepatocellular carcinoma. J Hepatol. (2015) 63:131–40. 10.1016/j.jhep.2015.02.031 25724366

[30] Hu Y Strong VEASO. Author reflections: quality of life after gastrectomy for cancer. Ann Surg Oncol. (2021) 28:57–8. 10.1245/s10434-020-09318-4 33169300

[31] Gigic B Nattenmuller J Schneider M Kulu Y Syrjala KL Bohm J. The role of CT-quantified body composition on longitudinal health-related quality of life in colorectal cancer patients: the Colocare study. Nutrients. 2020 12:1247. 10.3390/nu12051247 32353960 PMC7282010

[32] Sheean P Gomez-Perez S Joyce C Vasilopoulos V Bartolotta MB Robinson P. Body composition, serum biomarkers of inflammation and quality of life in clinically stable women with estrogen receptor positive metastatic breast cancer. Nutr Cancer. (2019) 71:981–91. 10.1080/01635581.2019.1595053 31037968

[33] Chaston TB Dixon JB. Factors associated with percent change in visceral versus subcutaneous abdominal fat during weight loss: findings from a systematic review. Int J Obes. (2008) 32:619–28. 10.1038/sj.ijo.0803761 18180786

[34] Dong QT Cai HY Zhang Z Zou HB Dong WX Wang WB. Influence of body composition, muscle strength, and physical performance on the postoperative complications and survival after radical gastrectomy for gastric cancer: a comprehensive analysis from a large-scale prospective study. Clin Nutr. (2021) 40:3360–9. 10.1016/j.clnu.2020.11.007 33223117

[35] Swisher AK Abraham J Bonner D Gilleland D Hobbs G Kurian S. Exercise and dietary advice intervention for survivors of triple-negative breast cancer: effects on body fat, physical function, quality of life, and adipokine profile. Support Care Cancer. (2015) 23:2995–3003. 10.1007/s00520-015-2667-z 25724409 PMC4624214

[36] Gudmundsson GH Johannsson E. Fitness, body composition and quality of life following cancer treatment. Laeknabladid. (2020) 106:179–85. 10.17992/lbl.2020.04.575 32234972

[37] Beaudart C Biver E Reginster JY Rizzoli R Rolland Y Bautmans I. Validation of the SarQoL(R), a specific health-related quality of life questionnaire for Sarcopenia. J Cachexia Sarcopenia Muscle. (2017) 8:238–44. 10.1002/jcsm.12149 27897430 PMC5377391

[38] Zou HB Yan XL Dong WX Yu DY Zhang FM Zhou LP. Sarcopenia is a predictive factor of poor quality of life and prognosis in patients after radical gastrectomy. Eur J Surg Oncol. (2021). 10.1016/j.ejso.2021.03.004 33714648

[39] Studenski S Perera S Wallace D Chandler JM Duncan PW Rooney E. Physical performance measures in the clinical setting. J Am Geriatr Soc. (2003) 51:314–22. 10.1046/j.1532-5415.2003.51104.x 12588574

[40] Guralnik JM Ferrucci L Pieper CF Leveille SG Markides KS Ostir GV. Lower extremity function and subsequent disability: consistency across studies, predictive models, and value of gait speed alone compared with the short physical performance battery. J Gerontol A Biol Sci Med Sci. (2000) 55:M221–31. 10.1093/gerona/55.4.M221 10811152 PMC12149745

[41] Guralnik JM Ferrucci L Simonsick EM Salive ME Wallace RB. Lower-extremity function in persons over the age of 70 years as a predictor of subsequent disability. N Engl J Med. (1995) 332:556–61. 10.1056/NEJM199503023320902 7838189 PMC9828188

[42] Biderman A Cwikel J Fried AV Galinsky D. Depression and falls among community dwelling elderly people: a search for common risk factors. J Epidemiol Community Health. (2002) 56:631–6. 10.1136/jech.56.8.631 12118057 PMC1732215

[43] Mishra SI Scherer RW Snyder C Geigle PM Berlanstein DR Topaloglu O. Exercise interventions on health-related quality of life for people with cancer during active treatment. Cochrane Database Syst Rev. (2012) 2012:CD008465. 10.1002/14651858.CD008465.pub2 22895974 PMC7389071

